# Long-COVID Syndrome? A Study on the Persistence of Neurological, Psychological and Physiological Symptoms

**DOI:** 10.3390/healthcare9050575

**Published:** 2021-05-13

**Authors:** Graziella Orrù, Davide Bertelloni, Francesca Diolaiuti, Federico Mucci, Mariagrazia Di Giuseppe, Marco Biella, Angelo Gemignani, Rebecca Ciacchini, Ciro Conversano

**Affiliations:** 1 Department of Surgical, Medical and Molecular Pathology and Critical Care Medicine, University of Pisa, Via Savi, 10, 56126 Pisa, Italy; davide.bertelloni@npmc.it (D.B.); francesca.diolaiuti@gmail.com (F.D.); mariagrazia.digiuseppe@gmail.com (M.D.G.); marco.biella@med.unipi.it (M.B.); angelo.gemignani@unipi.it (A.G.); rebecca.ciacchini@med.unipi.it (R.C.); ciro.conversano@unipi.it (C.C.); 2 Department of Biotechnology, Chemistry and Pharmacy, University of Siena, 53100 Siena, Italy; federico.mucci@med.unipi.it

**Keywords:** long COVID, long-haul COVID, quality of life, insomnia, COVID-19

## Abstract

Background: Emerging aspects of the Covid-19 clinical presentation are its long-term effects, which are characteristic of the so-called “long COVID”. The aim of the present study was to investigate the prevalence of physical, psychological, and sleep disturbances and the quality of life in the general population during the ongoing pandemic. Methods: This study, based on an online survey, collected demographic data, information related to COVID-19, sleep disturbances, and quality of life data from 507 individuals. The level of sleep disturbances and quality of life was assessed through the Insomnia Severity Index (ISI) and the EuroQol-5D (EQ-5D), respectively. Results: In total, 507 individuals (M = 91 and F = 416 women) completed the online survey. The main symptoms associated with “long COVID” were headache, fatigue, muscle aches/myalgia, articular pains, cognitive impairment, loss of concentration, and loss of smell. Additionally, the subjects showed significant levels of insomnia (*p* < 0.05) and an overall reduced quality of life (*p* < 0.05). Conclusions: The results of the study appear in line with recent publications, but uncertainty regarding the definition and specific features of “long COVID” remains. Further studies are needed in order to better define the clinical presentation of the “long COVID” condition and related targeted treatments.

## 1. Introduction

As of March 2021, it appears no longer necessary to repeat how and to what extent the COVID-19 pandemic has plagued humanity in the last year, being responsible for morbidity and mortality levels with few precedents in the recent history [1,2,3]. During the first weeks, in which we were all unprepared to face such a disaster, our attention had necessarily to be directed towards the negative and significant impact of the ongoing infection. 

Although most individuals initially developed a mild COVID-19 infection that did not require hospitalization [4], countless individuals needed to be admitted to intensive care units, experiencing life-threatening symptoms. A growing number of studies have reported a set of neurological complications [5,6,7,8] associated with COVID-19 and significant psychopathological symptoms related to intense distress (i.e., post-traumatic stress disorder, secondary traumatic stress, complicated grief and anxiety, amongst others) in the general population and health-care workers [9,10,11,12,13,14,15]. This corollary of symptoms is particularly exacerbated within frontline health-care workers, a category of professionals who are at a significant risk of experiencing high levels of burnout and compassion fatigue compared to the general population [16,17,18] and are in contact with individuals at the final stage of life in their clinical practice [19].

Emerging aspects of COVID-19 clinical presentation are the long-term effects, which, in the absence of any formally agreed definitions, characterize the so-called “*long-COVID*” or “*long-haul COVID*” described in recent international journals [20,21,22].

As highlighted by Marshall [20] in Nature, “People with more severe infections might experience long-term damage not just in their lungs, but in their heart, immune system, brain and elsewhere”. In this context, several patients are experiencing a vast variety of symptoms that persist after recovery, despite a negativized pharyngeal swab [23,24,25].

In order to track possible long-term effects caused by COVID-19 with the aim to improve health outcomes, a new initiative has been established in the United Kingdom, called the Post-Hospitalisation COVID-19 Study (PHOSP-COVID) (https://www.phosp.org/, accessed date 2 April 2021). PHOSP-COVID is a consortium of leading researchers and clinicians which aims to recruit 10,000 patients for a year, with the aim to analyze clinical factors derived from clinical assessments and gain a comprehensive understanding of COVID-19 long-term effects along with the patients’ medical, psychological, and rehabilitation needs, enabling, in this way, their full recovery.

Long COVID may incorporate the following symptoms: (i) cardiovascular (i.e., chest tightness, chest pain, palpitations); (ii) respiratory (i.e., breathlessness, cough); (iii) neurological (i.e., stroke, encephalopathy, meningoencephalitis, seizures, cognitive impairment, headache, sleep disturbance, dizziness, delirium); (iv) gastrointestinal (i.e., abdominal pain, nausea, diarrhea, anorexia and reduced appetite); (v) musculoskeletal (i.e., joint pain, muscle pain); (vi) inflammatory (i.e., fatigue, fever, pain); (vii) generalized and aspecific (i.e., skin rashes, tinnitus, earache, sore throat, dizziness, loss of taste and/or smell) (NICE guideline, 2020). Moreover, a few studies also outlined how the sleep patterns change in patients with long COVID, with a drastic reduction in sleep quantity and quality [26,27,28]. In a clinical research conducted by Kokou-Kpolou and colleagues in 2020 [29], the authors analyzed the prevalence of insomnia in the general population and its contributing factors in a sample of French individuals. According to the aim of the study, participants underwent a qualitative sleep assessment based on the Insomnia Severity Index (ISI), and the study confirmed that COVID-19-related worries and loneliness may represent the major contributing factor to clinical insomnia. All these mentioned symptoms can cause a dramatic decrease of the quality of life [30,31]. 

In the recent literature, when symptoms persist beyond 3 or 12 weeks, the resulting pathological conditions have been defined as “*long COVID/post-acute COVID*” and “*chronic post-COVID syndrome*”, respectively. Both these conditions are characterized by a multisystem syndrome that requires an integrated multidisciplinary intervention capable of dealing with both the residual physical symptoms and the consequent social discomforts and mental disorders [32].

In Italy, most COVID-19 patients presented evident symptoms (71.4% of the 31,845 confirmed cases as of 3 June 2020) related to the long-COVID condition. However, there is still limited evidence of this as yet, indeed there are no precise data regarding the persistence of symptoms or its long-term consequences in the weeks and months following the remission period [33]. Consequently, little is known or published on the supposed long-COVID syndrome and its long-term consequences. Some studies have reported that fatigue and headache are the main symptoms displayed by these individuals [25,27,34]. Other clinical studies reported increased sleep-related issues, including insomnia, and a deterioration of the life quality [25,27].

Furthermore, it is also necessary to underline that lung diseases are an established and well-known causes of fatigue [35], and the central nervous origin of post-viral fatigue should also be considered (i.e., inflammatory-mediated and neurocognitive dysfunction) when we analyze long-COVID-related symptoms. 

The purpose of the present study was to evaluate the physical and psychological health conditions of a representative sample of the Italian population suffering from symptoms related to long COVID. In particular, we focused our attention on the prevalence of physical, psychological, or neurological problems, insomnia, and the level of quality of life in the general population.

## 2. Materials and Methods

### 2.1. Participants

From 5 February to 15 February 2021, we collected 517 responses from individuals living in Italy through an online survey. Of these, we excluded two respondents younger than 18 years and eight additional individuals who did not give informed consent. The final sample consisted of 507 subjects, composed of 91 men (17.95%) and 416 women (82.05%) (Table 1).

The exclusions criteria were: minors, those who had not given informed consent, and non-native speakers of Italian.

### 2.2. Materials

Subjects were asked personal information and questions related to COVID-19, such as: “*Have you ever been affected by COVID-19?*”, “*Which of the following symptoms have you experienced during the last week?*”. We also administered questionnaires to assess insomnia measured by The Insomnia Severity Index (ISI) [36] and quality of life through the EuroQol-5D (EQ-5D) [37].

The Insomnia Severity Index (ISI) is a self-report tool that measures the perception that a subject has of both nocturnal and daytime symptoms of insomnia. It is composed of 7 items that evaluate the subjective perception of the difficulty in falling asleep, staying asleep and waking up early in the morning, the satisfaction or lack of it with the current sleep pattern, interference with daily functioning, as well as the evident impairment attributed to sleep problems and the degree of distress or worry caused by the reduction in sleep. Subjects must indicate the most appropriate answer on a 5-point Likert scale (0 “*not at all*” and 4 “*very much*”). The sum of the scores indicates the level of insomnia. From 0 to 7, there is no clinically significant insomnia; from 8 to 14, insomnia is below the clinical threshold; from 15 to 21, there is clinical insomnia (medium severity); from 22 to 28, clinical insomnia is severe.

The EuroQol-5D (EQ-5D) is a self-report instrument for measuring the quality of life. EQ-5D was introduced by the EuroQol Group (1990) and allows to assess the general level of health. It has two main components: health state description and evaluation. In the first component, five dimensions are measured, which are mobility, self-care, usual activities, pain/discomfort, and anxiety/depression. Subjects must respond on five items (1 for each dimension) on a 3-point Linkert scale. From this measure, it is possible to obtain a health profile of the subject (EQ-5D-3L). The second component is called Visual Analogue Scale (EQ-VAS). Subjects are asked to indicate their state of health on a vertical line ranging from 0 to 100, where 0 is the worst health level imaginable, and 100 is the best health level imaginable.

### 2.3. Procedures

An online survey was launched on 5 February at 9:00 (GMT + 1). Respondents were recruited through Facebook, Linkedin, or via a direct link sent by e-mail. The participants were informed about the present study’s purposes and were asked to give their consent on personal data treatment. All procedures followed the ethical standards and were approved by the Ethics Committee of the University of Pisa (n. 0036344/2020).

### 2.4. Statistical Analysis

Descriptive statistics were used to examine the data collected. The data presented indicate the number and the percentage of participants who present a certain variable, in relation to the totality of the sample. We then calculated the incidence (percentage) of physical and psychological status. Means, percentages, and *T*-test were used to analyze the scores of the EQ-5D and ISI questionnaires.

## 3. Results

Table 1 shows descriptive statistics for socio-demographic characteristics and participants’ status (COVID-19-positive or not). Descriptive data are presented as number (N) and percentage (%) of subjects belonging to the group.

**Table 1:** Demographic characteristics.

	Group	N	%
Sex	Male	91	17.95%
	Female	416	82.05%
	Tot.	507	100%
Age	<20	1	0.20%
	20–29	62	12.23%
	30–39	106	20.91%
	40–49	156	30.77%
	50–59	132	26.04%
	60–69	42	8.28%
	>70	8	1.58%
Educational Level	Elementary, middle, and high school education	283	55.82%
	Graduates, doctoral students, and master students	224	44.18%
COVID-19 status	I’ve never been tested positive	33	6.51%
	I am currently positive for COVID-19	20	3.94%
	I had COVID-19 but have not been positive (swab result: negative) for less than a month	68	13.41%
	I had COVID-19 but have not been positive (swab result: negative) for at least a month	74	14.60%
	I had COVID-19 but have not been positive (swab result: negative) for at least two months	154	30.37%
	I had COVID-19 but have not been positive (swab result: negative) for at least three months	152	29.98%
	Other	6	1.18%
Cases of COVID-19 within the family	none	74	14.60%
	1 case	133	26.23%
	2 cases	121	23.87%
	>3 cases	179	35.31%

### 3.1. Symptoms Long-COVID-19 Syndrome

Participants indicated their COVID-19 status as follows: “I have never been positive for COVID-19”; “I am currently positive for COVID-19”; “I had COVID-19 but have not been positive (swab result: negative) for less than a month”; “I had COVID-19 but have not been positive (swab result: negative) for at least a month”; “I had COVID-19 but have not been positive (swab result: negative) for at least two months”; “I had COVID-19 but have not been positive (swab result: negative) for at least three months”; “Other”. 

**Figure 1:**
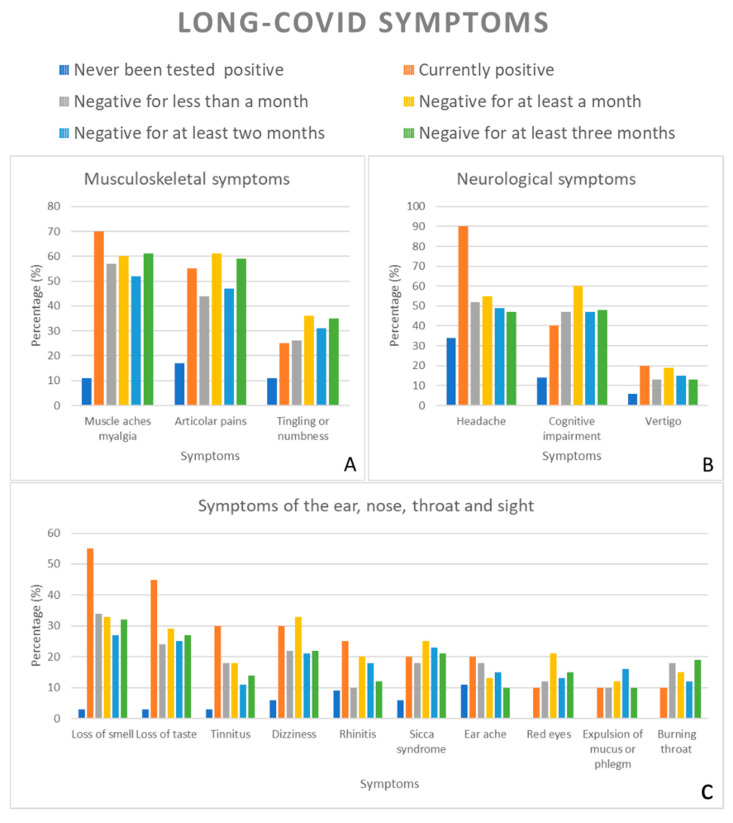

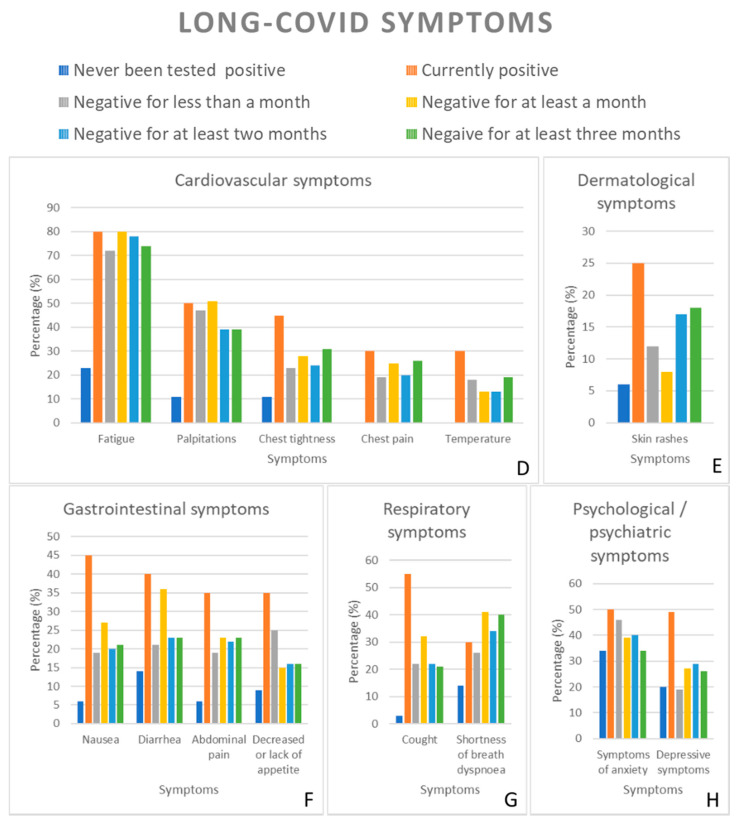
Percentages of symptoms reported by subjects in different COVID-19 conditions. The symptoms were divided into sub-categories: Musculoskeletal symptoms (**A**); Neurological symptoms (**B**); Symptoms regarding the ear, nose, throat, and sight (**C**); Cardiovascular symptoms (**D**); Dermatological symptoms (**E**); Gastrointestinal symptoms (**F**); Respiratory symptoms (**G**); Psychological/Psychiatric symptoms (**H**).

We then asked participants to report the symptoms they experienced in the past 7 days. Table 2 and Figure 1 show the incidence of the single symptoms listed in the questionnaire divided according to the indicated positivity status. The proposed symptoms were identified according to the guidelines for COVID-19 and long COVID-19 reported by the National Institute for Health and Care Excellence (NICE), Royal College of General Practitioners (RCGP), and Healthcare Improvement Scotland (SIGN) [38].

The data show that the main symptoms reported by subjects, currently suffering from COVID-19 (swab positive) were: (a) headache (90%); (b) fatigue (80%); (c) muscle aches/myalgia (70%); (d) articular pains (55%); (e) loss of smell (55%). On the other hand, subjects who were positive but had been negative for less than a month reported the following symptoms: fatigue (72%), muscle aches/myalgia (57%), and headache (53%). The symptoms reported by subjects that previously tested positive for COVID-19 but at the time of completing the questionnaire had been negative for more than a month were: fatigue (80%), articular pains (61%), muscle aches/myalgia (59%), loss of concentration (59%), and headache (54%). Similarly, subjects who previously tested positive for COVID-19 but had been negative for more than 2 months reported the following symptoms: fatigue (79%), muscle aches/myalgia (53%), and headache (49%). Finally, individuals who had tested negative for COVID-19 for at least 3 months indicated that they suffered from these symptoms: fatigue (74%), muscle aches/myalgia (61%), and articular pains (59%).

In conclusion, the most reported symptoms by subjects previously positive for COVID-19 but currently negative were the following: headache, fatigue, muscle aches/myalgia, articular pains, cognitive impairment and loss of concentration, and loss of smell. The prevalence of these symptoms shows a decreasing trend o over time. For example, positive subjects that tested negative for more than 3 months reported a lower incidence of symptoms compared to positive subjects that tested negative for less than a month.

### 3.2. Insomnia and COVID-19

**Figure 2:**
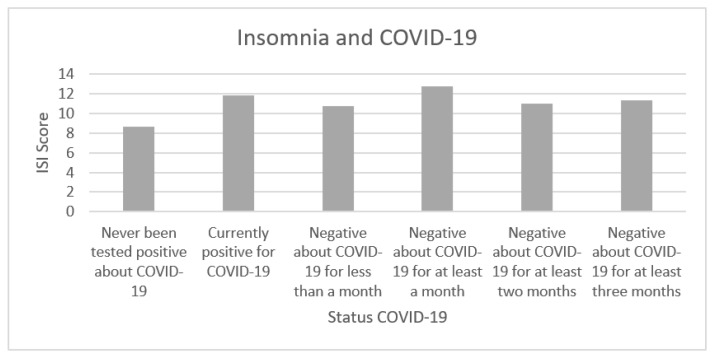
The mean values of the scores indicated by the subjects regarding their insomnia (ISI score) are reported, stratified by COVID-19 status.

We investigated whether insomnia, measured by ISI, presented a higher incidence in subjects who currently had COVID-19 and subjects who had COVID-19 in the past but at the time of the survey tested negative, compared to subjects who never tested positive for COVID-19. To ensure that our ISI measurement was reliable, we computed the Cronbach’s alpha, which reached a satisfactory level (α = 0.87). Table 3 and Figure 2 show the average scores reported by questionnaire participants classified according to their COVID-19 status.

To compare the differences between ISI average scores in relation to the COVID-19 status, we carried out Welsch’s *T*-Tests which do not assume equal variances between the groups and are more robust for unbalanced sample sizes. We investigated whether there were differences between subjects who were currently positive for COVID-19 or who had been positive in the past but currently tested negative for the swab (had been negative for different times from the last negative swab) and subjects who never had COVID-19. To keep the type I error under control, all presented *p*-values were adjusted to multiple comparisons (false discovery rate). Table 4 shows significant differences between the group of subjects who never had COVID-19 and subjects with the following conditions: negative for COVID-19 for less than a month (*t* (55.64) = 1.63; *p* = 0.054); negative for COVID-19 for at least a month (*t* (49.60) = 3.32; *p* = 0.004); negative for COVID-19 for at least two months (*t* (44.36) = 1.95; *p* = 0.046); negative for COVID-19 for at least three months (*t* (44.94) = 2.24; *p* = 0.037).

### 3.3. Long-COVID-19 Syndrome and Quality of Life

The EQ-5D data (Table 5) showed that subjects with COVID-19 and long COVID-19 had serious problems in carrying out daily activities. It is striking to note the different prevalence (in percentage) of the mentioned problems in subjects currently positive for COVID-19 (15%) and in subjects no longer positive for COVID-19 (10.29% for less than a month; 9.46% for at least a month; 3.90% for at least two months; 3.95% for at least three months), compared to subjects who had never been positive for COVID-19 (0%). Subjects who previously tested positive for COVID-19 but at the time of the survey were negative, reported moderate and severe levels of pain and discomfort (75% of subjects negative less than a month; 83.78% for at least a month; 81.17% for at least two months; 79.6% for at least three months). These levels were higher than those of subjects who had COVID-19 (30%) at the time of the survey and for subjects who had never had COVID-19 (36.36%).

**Figure 3:**
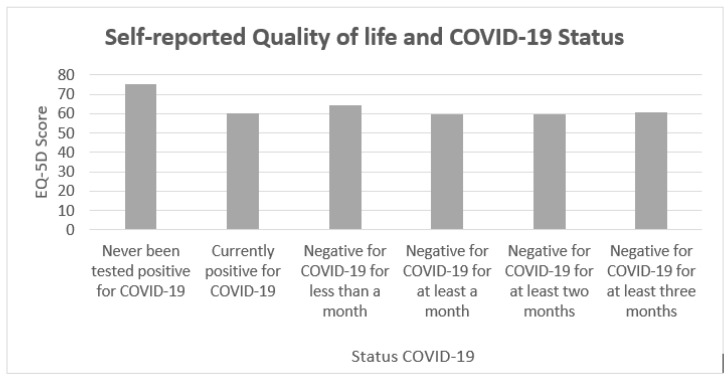
Mean values of the scores indicated by the subjects regarding their quality of life, classified according to the COVID-19 status.

Moreover, when subjects were asked to indicate a value from 1 to 100 indicating their quality of life, individuals who had never been positive for COVID-19 showed mean values (M = 75.54) greater than those of subjects who had COVID-19 at that time of the survey (M = 60.54; *p* < 0.05) and of subjects who had had COVID-19 but were negative at that time of the survey (*p* < 0.05) (Table 6, Figure 3). Subjects with COVID-19 and symptoms of long COVID-19 reported on average a level of quality of life 14.64 points lower (19.5% less; *p* < 0.05) compared to subjects who never had COVID-19.

## 4. Discussion

Our results remark that the long-COVID-19 syndrome encompasses a rather heterogeneous set of symptoms (including headache, fatigue, muscle aches/myalgia, articular pains, cognitive impairment and loss of concentration, and loss of smell) that, however, appear to recede over time. In addition, when taking into account sleep disturbances, our results highlight that patients with COVID-19 and persistent symptoms associated with the long-COVID-19 condition showed a significantly higher rate of insomnia than subjects who never had COVID-19 (*p* < 0.05).

As for the psychological characteristics, higher levels of pain and discomfort, anxiety, and/or depression were observed in patients with long COVID-19 than in those who never had COVID-19. On the other hand, patients currently positive for COVID-19 and with long-COVID-19 symptoms reported lower quality of life compared to subjects who had never tested positive for COVID-19.

Such findings appear in line with those reported by other studies and therefore seem to confirm the results currently available in the scientific literature [25,27,34]. In the recent study conducted by Carfì and colleagues [25], 143 subjects were evaluated after 60 days from the onset of COVID-19. Of these, only 18 (12.6%) appeared completely asymptomatic, while 32% presented one or two symptoms, and 55% presented three or more. More specifically, 53.1% of participants reported fatigue, 43.4% dyspnea, 27.3% joint pain, and 21.7% chest pain. Such results appear substantially in line with those observed by Mandal and colleagues [34], indicating that subjects reported a persistence of fatigue (69%), dyspnea (53%), and cough (34%) after 54 days from their negative swab. Finally, a sample of 1733 subjects who had COVID-19 was re-evaluated on average 186 days after the negative swab, and muscle fatigue or weakness was observed in 63% of them.

As initially hypothesized, our results confirmed that patients with COVID-19 or long-COVID-19 syndrome had significantly higher rates of insomnia than subjects who never had COVID-19. Interestingly, such findings are very much similar to those reported in a recent study, in which 26% of participants with long-COVID-19 syndrome showed sleep disturbances [27]. It could also be speculated that the quality of sleep might be negatively affected by the overall pandemic distressful situation as well as by the national security measures applied to contain the spread of the infection. However, our control group (namely, respondents who never tested positive for COVID-19) was exposed to the same containment strategies and social situations, somehow ruling out this alternative explanation. Crucially, our results pointing toward increased sleep disturbance and increased level of mental distress (i.e., anxiety) converge with those of a recent investigation by Alaly and Bowe [39], which documented increased hazard ratio for those disturbances. (HR_sleep-disorders_ = 14.53, HR_anxiety_ = 5.42) and for death (HR_death_ = 1.59) after 6 months from the infection.

Finally, subjects with COVID-19 and long-COVID-19 showed a self-reported level of quality of life that was 14.64 points lower (19.5% less) than that of subjects who never had COVID-19. These results are consistent with the outcomes reported in recent investigations that revealed a reduction in the quality of life of subjects with COVID-19 or long-COVID syndrome [25,27].

This study suffers from a number of limitations: (1) the COVID-19 groups were not homogeneous; therefore, future works will have to investigate larger samples and provide statistically homogeneous data; (2) it is advisable to investigate other types of physical and psychological symptoms and to explore whether these are specifically related to COVID-19 or long-COVID-19 syndrome or if this symptomatology is present in other clinical populations; (3) the recruitment channels used could have led us to bias; therefore, future studies will need to expand and balance the recruitment channels; (4) at the time of the survey, the Italian regions were classified according to the level of infection and subjected to different containment measures, put in place by the Italian legislation; unfortunately, this study did not investigate the impact of the different measures of containment, and future studies should take in account such differences; (4) given the nature of this investigation, the study did not explore premorbid conditions of the sample.

As suggested by different empirical studies, there is a great utility in focusing on prediction, rather than explanation, during data analysis, both in the clinical and in the social sciences settings [37,40,41].

In conclusion, the present study highlights that the main symptoms associated with the so-called long-COVID-19 syndrome are the following: headache, fatigue, muscle aches/myalgia, articular pains, cognitive impairment and loss of concentration, and loss of smell. Additionally, in the analyzed sample, insomnia and a reduced quality of life were detected. Due to the limitations of the present investigation, the challenges encountered, and the limited number of studies available so far, robust conclusions are precluded. Further studies are needed to better define the clinical presentation of the long-COVID condition and consequently related tailored treatments.

## Figures and Tables

**Table 2:** Number (N) and percentages (%) of the subjects who indicated a set of symptoms, stratified by COVID-19 positivity status.

Symptoms	Have You Ever Been Affected by COVID-19?						
	I’ve Never Been Tested Positive for COVID-19	I Am Currently Positive for COVID-19	I Had COVID-19 But Have Not Been Positive (Swab Result: Negative) for Less Than a Month	I Had COVID-19 But Have Not Been Positive (Swab Result: Negative) for at Least a Month	I Had COVID-19 But Have Not Been Positive (Swab Result: Negative) for at Least Two Months	I Had COVID-19 But Have Not Been Positive (Swab Result: Negative) for at Least Three Months	Other
Shortness of breath (dyspnea)	5 (15.15%)	6 (30%)	18 (26.47%)	30 (40.54%)	52 (33.77%)	61 (40.13%)	3 (50%)
Cough	1 (3.03%)	11 (55%)	15 (22.06%)	23 (31.08%)	35 (22.73%)	32 (21.05%)	1 (16.67%)
Chest tightness	4 (12.12%)	9 (45%)	16 (23.53%)	21 (28.38%)	38 (24.68%)	48 (31.58%)	3 (50%)
Chest pain	0 (0%)	6 (30%)	13 (19.12%)	19 (25.68%)	31 (20.13%)	40 (26.32%)	3 (50%)
Palpitations	4 (12.12%)	10 (50%)	32 (47.06%)	37 (50%)	60 (38.96%)	59 (38.82%)	2 (33.33%)
Headache	12 (36.36%)	18 (90%)	36 (52.94%)	40 (54.05)	76 (49.35%)	71 (46.71%)	2 (33.33%)
Fatigue	8 (24.24%)	16 (80%)	49 (72.06%)	59 (79.73%)	121 (78.57%)	113 (74.34%)	6 (100%)
Temperature	0 (0%)	6 (30%)	12 (17.65%)	9 (12.16%)	19 (12.34%)	29 (19.07%)	1 (16.67%)
Cognitive impairment (brain fog, loss of concentration)	5 (15.15%)	8 (40%)	32 (47.06%)	44 (59.46%)	73 (47.40%)	74 (48.68%)	6 (100%)
Tingling or numbness	4 (12.12%)	5 (25%)	18 (26.47%)	26 (35.14%)	48 (31.17%)	53 (34.87%)	2 (33.33%)
Dizziness	2 (6.06%)	6 (30%)	15 (22.06%)	25 (33.78%)	33 (21.43%)	34 (22.37%)	1 (16.67%)
Abdominal pain	2 (6.06%)	7 (35%)	13 (19.12%)	17 (22.97%)	35 (22.73%)	35 (23.03%)	2 (33.33%)
Nausea	2 (6.06%)	9 (45%)	13 (19.12%)	19 (25.68%)	30 (19.48%)	31 (20.39%)	3 (50%)
Diarrhea	5 (15.15%)	8 (40%)	14 (20.59%)	27 (36.49%)	36 (23.38%)	34 (22.37%)	1 (16.67%)
Decreased or lack of appetite	3 (9.09%)	7 (35%)	17 (25%)	10 (13.61%)	24 (15.58%)	24 (15.79%)	0 (0%)
Articular pains	6 (18.18%)	11 (55%)	30 (44.12%)	45 (60.81%)	73 (47.40%)	90 (59.21%)	3 (50%)
Muscle aches (myalgia)	4 (12.12%)	14 (70%)	39 (57.35%)	44 (59.46%)	81 (52.60%)	93 (61.18%)	5 (83.33%)
Depressive symptoms	7 (21.21%)	8 (49%)	13 (19.12%)	20 (27.03%)	46 (29.87%)	39 (25.66%)	3 (50%)
Symptoms of anxiety	11 (33.33%)	10 (50%)	31 (45.59%)	28 (37.84%)	62 (40.26%)	52 (34.21%)	2 (33.33%)
Earache	4 (12.12%)	4 (20%)	12 (17.65%)	10 (13.61%)	24 (15.58%)	15 (9.87%)	0 (0%)
Expulsion of mucus or phlegm from the nose and/or mouth	0 (0%)	2 (10%)	7 (10.29%)	9 (12.16%)	24 (15.58%)	15 (9.87%)	0 (0%)
Rhinitis (runny nose and nasal obstruction)	3 (9.09%)	5 (25%)	7 (10.29%)	15 (20.27%)	28 (18.18%)	18 (11.84%)	1 (16.67%)
Sicca syndrome (the most common eye symptoms are dryness, foreign body and burning sensation)	2 (6.06%)	4 (20%)	12 (17.65%)	19 (25.68%)	36 (23.38%)	32 (21.05%)	1 (16.67%)
Tinnitus (ringing in the ear)	1 (3.03%)	6 (30%)	12 (17.65%)	13 (17.57%)	17 (11.04%)	22 (14.47%)	0 (0%)
Burning throat	0 (0%)	2 (10%)	12 (17.65%)	11 (14.86%)	18 (11.69%)	27 (17.76%)	0 (0%)
Loss of taste	1 (3.03%)	9 (45%)	16 (23.53%)	21 (28.38%)	39 (25.32%)	41 (26.97%)	2 (33.33%)
Loss of smell	1 (3.03%)	11 (55%)	23 (33.82%)	24 (32.43%)	42 (27.27%)	47 (30.92%)	2 (33.33%)
Red eyes	0 (0%)	2 (10%)	8 (11.76)	16 (21.62%)	21 (13.64%)	24 (15.79%)	0 (0%)
Skin rashes	2 (6.06%)	5 (25%)	8 (11.76)	6 (8.11%)	26 (16.88%)	28 (18.42%)	1 (16.67%)
Vertigo	2 (6.06%)	4 (20%)	9 (13.24%)	13 (17.57%)	22 (14.29%)	20 (13.16%)	1 (16.67%)
Anything	13 (39.39%)	0 (0%)	3 (4.41%)	0 (0%)	3 (1.95%)	4 (2.63%)	0 (0%)
Other	1 (3.03%)	3 (15%)	12 (17.65%)	8 (10.81%)	17 (11.04%)	21 (13.82%)	1 (16.67%)

**Table 3:** Mean values (M) and standard deviation (SD) of the ISI scores stratified according to the different COVID-19 conditions.

Status Positivity COVID-19	ISI Score
	M (SD)
Never been tested positive for COVID-19	8.67 (6.37)
Currently positive for COVID-19	11.85 (5.98)
Negative for COVID-19 for less than a month	10.78 (5.48)
Negative for COVID-19 for at least a month	12.81 (4.90)
Negative for COVID-19 for at least two months	11.01 (5.86)
Negative for COVID-19 for at least three months	11.38 (5.95)
Other	14 (3.63)

**Table 4:** T-Test analysis (*p* < 0.05, FDR) of IRI Scores between “never been positive for COVID-19” and “currently positive for COVID-19 and negative for COVID-19 for less than a month”, “currently positive for COVID-19 and negative for COVID-19 for at least a month”, “currently positive for COVID-19 and negative for COVID-19 for at least two months”, “currently positive for COVID-19 and negative for COVID-19 for at least three months” and “other”.

T-Value Status COVID-19	Never Been Tested Positive about COVID-19
Currently positive for COVID-19	1.80
Negative for COVID-19 for less than a month	1.63
Negative for COVID-19 for at least a month	3.32 **
Negative for COVID-19 for at least two months	1.95 *
Negative for COVID-19 for at least three months	2.24 *
Other	1.98

* < 0.05, ** < 0.01.

**Table 5:** Number and percentage of subjects who responded to individual items in the five dimensions investigated by the EQ-5D, classified by COVID-19 Status and EQ-5D Scale.

EQ-5D Scales	Severity	Status Positivity COVID-19						
		Never Tested Positive for COVID-19 N (%)	Currently Positive for COVID-19 N (%)	Negative for COVID-19 for Less Than a Month N (%)	Negative for COVID-19 for at Least a Month N (%)	Negative for COVID-19 for at Least Two Months N (%)	Negative for COVID-19 for at Least Three Months N (%)	Other N (%)
Movement ability	No problem	29 (87.88%)	15 (75%)	53 (77.94%)	43 (58.11%)	110 (71.43%)	115 (75.66%)	4 (66.67%)
	Moderate Problems	4 (12.12%)	4 (20%)	15 (22.06%)	31 (41.89%)	43 (27.92%)	37 (24.34%)	2 (33.33%)
	Serious problems	0 (0%)	1 (5%)	0 (0%)	0 (0%)	1 (0.65%)	0 (0%)	0 (0%)
Self-care	No problem	30 (90.91%)	18(90%)	63 (92.65%)	66 (89.19%)	141 (91.56%)	136 (89.47%)	6 (100%)
	Moderate Problems	3 (9.09%)	2 (10%)	5 (7.35%)	8 (10.81%)	12 (7.79%)	15 (9.87%)	0 (0%)
	Serious problems	0 (0%)	0 (0%)	0 (0%)	0 (0%)	1 (0.65%)	1 (0.66%)	0 (0%)
Daily activities	No problem	20 (60.61%)	8 (40%)	30 (44.12%)	48 (64.86%)	53 (34.41%)	48 (31.58%)	3 (50%)
	Moderate Problems	13 (39.39%)	9 (45%)	31 (45.59%)	19 (25.68%)	95 (61.69%)	98 (64.47%)	3 (50%)
	Serious problems	0 (%)	3 (15%)	7 (10.29%)	7 (9.46%)	6 (3.90%)	6 (3.95%)	0 (0%)
Pain, discomfort	No problem	21 (63.64%)	14 (70%)	17 (25%)	12 (16.22%)	29 (18.83%)	31 (20.39%)	2 (33.33%)
	Moderate Problems	10 (30.30%)	4 (20%)	48 (70.59%)	51 (68.92%)	113 (73.38%)	109 (71.71%)	4 (66.67%)
	Serious problems	2 (6.06%)	2 (10%)	3 (4.41%)	11 (14.86%)	12 (7.79%)	12 (7.89%)	0 (0%)
Anxiety and/or Depression	No problem	9 (27.27%)	8 (40%)	26 (38.24%)	24 (32.43%)	51 (33.12%)	60 (39.47%)	3 (50%)
	Moderate Problems	19 (57.58%)	8 (40%)	37 (54.41%)	39 (52.70%)	90 (58.44%)	76 (50%)	3 (50%)
	Serious problems	5 (15.15%)	4 (20%)	5 (7.35%)	11 (14.86%)	13 (8.44%)	16 (10.53%)	0 (0%)

**Table 6:** Mean values (M) and standard deviation (SD) for subjects who responded to individual items in the five dimensions investigated by the EQ-5D, classified by COVID-19 status and EQ-5D Scale.

Positivity for COVID-19	EQ-5D Score
	M (SD)
Never been tested positive for COVID-19	75.54 (17.09)
Currently positive for COVID-19	60.45 (17.18)
Negative for COVID-19 for less than a month	64.59 (18.88)
Negative for COVID-19 for at least a month	59.85 (16.88)
Negative for COVID-19 for at least two months	59.65 (16.52)
Negative for COVID-19 for at least three months	60.85 (19.96)
Other	60 (19.71)

## Data Availability

The data presented in this study are available on request from the corresponding author. The data are not publicly available due to privacy issue.
